# Mode of Action of Shan-Zhu-Yu (*Cornus officinalis* Sieb. et Zucc.) in the Treatment of Depression Based on Network Pharmacology

**DOI:** 10.1155/2020/8838888

**Published:** 2020-11-22

**Authors:** Ping Liu, Ping Yang, Lan Zhang

**Affiliations:** ^1^Department of Pharmacy, Xuanwu Hospital of Capital Medical University, National Clinical Research Center for Geriatric Diseases, Beijing Engineering Research Center for Nervous System Drugs, Beijing Institute for Brain Disorders, Key Laboratory for Neurodegenerative Diseases of Ministry of Education, Beijing 100053, China; ^2^Department of Clinical Pharmacy, Key Laboratory of Basic Pharmacology of Guizhou Province and School of Pharmacy, Zunyi Medical University, Zunyi, Guizhou 563000, China

## Abstract

**Background:**

Although the traditional Chinese medicine Shan-Zhu-Yu may be efficacious against depression, its mechanism of action is unknown. In this study, we aimed to explore the possible mechanisms of action of Shan-Zhu-Yu in the treatment of depression using network pharmacology.

**Methods:**

The active ingredients and targets of Shan-Zhu-Yu were obtained from the Traditional Chinese Medicine System Pharmacology Database (TCMSP) database and converted into gene names using UniProt. Then, the target genes of depression were collected using GeneCards and OMIM. Drug disease intersection genes were obtained using a Venn tool, and a protein-protein interaction network was constructed using STRING. Cytoscape was used to construct an active ingredients-targets-drug-disease network. GO and KEGG pathway enrichment analyses were performed using DAVID. Furthermore, Autodock was used to evaluate drug and target binding and explore possible molecular mechanisms.

**Results:**

We identified 9721 disease genes, 13 active ingredients, 50 target genes, and 48 drug disease intersecting genes. The results of the GO enrichment analysis suggested that Shan-Zhu-Yu affects the activity of G protein-coupled amine, neurotransmitter, steroid hormone, nuclear, and G protein-coupled neurotransmitter receptors in the treatment of depression by acting on hormone and nuclear receptor binding. The main signaling pathways were associated with neuroactive ligand-receptor interaction, calcium, cGMP-PKG, apoptosis, estrogen, p53, and AGE-RAGE. Molecular docking confirmed that the active components of Shan-Zhu-Yu (e.g., telocinobufagin and *β*-sitosterol) docked suitably with *NR3C1*, *Bax*, *Bcl-2*, and *caspase-3*. Shan-Zhu-Yu may exert its therapeutic effects on depression via multiple targets and pathways.

**Conclusions:**

The present study elucidates that Shan-Zhu-Yu suppresses the expression of *Bax* and *caspase-3* and promotes that of *NR3C1* and *Bcl-2* through neuroactive ligand-receptor interaction and apoptosis signaling pathways. Therefore, Shan-Zhu-Yu is a potential treatment option for depression, and the results of this study will provide new reference points for future experimental research and a scientific basis for its widespread clinical application.

## 1. Introduction

Depression, a mood or affective mental disorder characterized by anxiety or depressive behaviors, can be caused by various factors. The main symptoms of depression include permanent low spirit, mental retardation, physical discomfort, decreased volitional activity, and cognitive impairment, and the main clinical manifestations are low mood, lack of pleasure, decreased self-worth, and even suicidal thoughts [1]. In addition, depression is a risk factor for diabetes [2] and cardiovascular disease [3]. Murray and Lopez [4] in a 4.5 year Global Burden of Disease study showed that unipolar major depression accounted for 3.7% of disability-adjusted life years (DALYs) in 1990, ranking the fourth, and predicted this to be up to 5.7% in 2020. In China, the prevalence of depression and DALY rates increased in all provinces [5]; however, the etiology and pathogenesis of depression remain unclear.

Depression, which is caused by a combination of genetic, environmental, and spiritual factors, involves neurotransmitters, inflammation, and hypothalamic pituitary adrenal (HPA) axis changes, among other processes [6]. Currently, negative treatment outcomes are common. The main clinical treatment of depression involves Western medicine, and the most widely used classic antidepressants are based on the “monoamine hypothesis” [7], which states that blocking the reuptake of catecholamine neurotransmitters at the presynaptic membrane effectively increases the concentration of catecholamine neurotransmitters in the synaptic cleft, thereby improving depressive symptoms. The antidepressants developed based on this theory include selective serotonin reuptake inhibitors and serotonin and norepinephrine reuptake inhibitors. Although these drugs are tolerated and cause only mild side effects, they have a single-target and slow onset [8]. Therefore, new effective drugs need to be developed; however, owing to the existence of various complex pathways and negative feedback regulation mechanisms in the nervous system [9], the development of more effective single-target drugs and monotherapies is difficult. In the future, multitarget and combination drugs will be essential in the treatment of depression [10].

Traditional Chinese medicine has unique advantages and potential in the treatment of depression. It has “theories” to understand the pathogenic mechanism and classify the syndrome [11]. Furthermore, traditional Chinese medicine, alone and in combination with Western medicine, has been shown to have a rapid onset, increase the cure rate, and reduce the incidence of side effects compared with Western medicine treatment alone; however, research on the mechanisms of action is warranted [12].

Shan-Zhu-Yu, the dried and mature pulp of *Cornus officinalis* Sieb. et Zucc., also known as fructus corni and jujube, is included in the Chinese Pharmacopoeia (2020), in sections related to the liver, kidney homeostasis, liver and kidney tonics, and astringent effects. This herb has a wide range of pharmacological activities, such as hepatic and renal protection, antidiabetic, cardioprotective, antioxidant, neuroprotective, antitumor, anti-inflammatory, analgesic, antiaging, antiamnesic, anti-osteoporotic, antidepressive, and immunoregulatory effects [13]. For example, fructus corni-containing Liuwei Dihuang pills have been shown to improve depressive symptoms, reduce the incidence of adverse reactions, and improve the quality of life in patients with depression compared with Western medicine treatment alone [14] and exert antidepressant effects in rats under chronic mild stress [15]. Nonetheless, there is still a lack of systematic studies on the use of Shan-Zhu-Yu for the treatment of depression-like symptoms and its mechanism of action.

Although the multitarget and multichannel approach of traditional Chinese medicine can provide new ideas for the treatment of clinically complex diseases, it also increases the difficulty in research. Network pharmacology, a new approach for drug design based on the rapid development of systems biology and multidirectional pharmacology, goes beyond the *single target*. In network pharmacology, a *multitarget* research strategy, which has scalability, effectiveness, and reliability [16], is implemented for drug discovery. It is widely used to determine disease targets, the clinical efficacy of compounds, the mechanism of action, and toxicity, as well as the material basis and mechanism of action of traditional Chinese medicine [17, 18]. In this study, network pharmacology was used to screen biomarkers of depression and predict the therapeutic targets of Shan-Zhu-Yu, in the hope of providing ideas for basic research and treatment of depression.

## 2. Materials and Methods

### 2.1. Screening of Active Compounds and Prediction of Putative Targets of Shan-Zhu-Yu

We searched the Traditional Chinese Medicine System Pharmacology Database (TCMSP, http://tcmspw.com/tcmsp.php) using “Shan-Zhu-Yu” as the key word, selected the active ingredients with an oral bioavailability (OB) ≥30% and drug-likeness (DL) ≥0.18, and obtained their corresponding targets using “Related Targets [19]”. Next, the target names were input into the Universal Protein Database (UniProt, https://www.uniprot.org/), and *Homo sapiens* was selected to normalize the gene information.

### 2.2. Identification of Depression-Related Targets

Online Mendelian Inheritance in Man (OMIM, https://omim.org/) and GeneCards (https://www.genecards.org/) were used to obtain the related depression targets, and the results were summarized to remove duplicates.

### 2.3. Filtering Intersecting Targets

We imported the depression targets and putative targets of Shan-Zhu-Yu into a Venn tool (http://bioinformatics.psb.ugent.be/webtools/Venn/) to obtain intersecting targets.

### 2.4. Protein-Protein Interaction (PPI) Construction

The intersecting targets of Shan-Zhu-Yu and depression were input into STRING (https://string-db.org/) to generate a protein-protein interaction network. The minimum interaction score was set to 0.70, and the nodes that were not connected with the main network were hidden. The TSV file format was downloaded to construct an active ingredients-targets-drug-disease network with Cytoscape 3.2.1 software; the network in STRING is the PPI.

### 2.5. Gene Ontology (GO) and Kyoto Encyclopedia of Genes and Genomes (KEGG) Pathway Enrichment Analyses

We imported intersecting targets into the functional annotation tool of the Database for Annotation, Visualization and Integrated Discovery (DAVID, https://david.ncifcrf.gov/) and used *R* 3.6.1 to produce simple and clear results [20].

### 2.6. Molecular Docking Simulation

#### 2.6.1. Ligand Preparation

We used the PubChem database (https://www.ncbi.nlm.nih.gov/pubmed) to collect and download the 2D structure of small molecule compounds. These were saved in Mol2 format after energy minimization in Chem3D and PDBQT formats after setting as spin by AutoDockTools (ADT).

#### 2.6.2. Target Protein Preparation

The crystal structures of nuclear receptor subfamily 3 group C member 1 (*NR3C1*) (PDBID: 1NHZ) and *caspase-3* genes (PDBID: 1NMS) were downloaded from the Protein Data Bank (http://www.rcsb.org/). As B-cell lymphoma 2 (Bcl-2) and Bcl-2-like protein 4 (*Bax*) have no crystal structure in the database, homology modeling was performed, UniProt was used to query the amino acid sequence, and the SWISS-MODEL server was used to model and evaluate quality. The downloaded complexes were embellished using PyMol1.7 to remove the original ligand and water molecules and saved in PDBQT format. Moreover, AutoDockTools-1.5.6 software was used to prepare receptors, including the addition of hydrogen and charge.

#### 2.6.3. Molecular Docking

The prepared files were imported into Discovery Studio 3.5 Client software to search for active pockets. Telocinobufagin was set to dock with *NR3C1* and *β*-sitosterol with *Bax*, *Bcl-2*, and *caspase-3*. The lowest energy conformation was selected as the optimal for analysis. PyMol was used for dock site analysis, and Discovery Studio was used to analyze the interaction force between small molecule ligands and amino acid residues.

## 3. Results

### 3.1. Shan-Zhu-Yu Target Predictions

**Figure 1:**
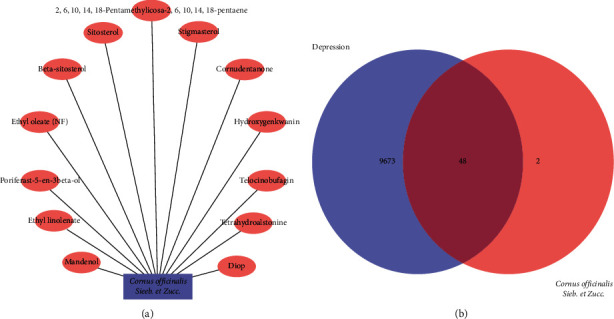
Shan-Zhu-Yu active ingredients and potential targets of treating depression. Shan-Zhu-Yu and its active ingredients (a). Intersecting genes Venn diagram of Shan-Zhu-Yu and treating depression (b).

OB, which is a measure of the pharmacokinetic process and druggability *in vivo*, and DL, which represents the similarity between unknown components and the known chemical structure of drugs, are important parameters to analyze the effectiveness of traditional Chinese medicine [21]. The study combined OB ≥30% and DL ≥0.18 and identified 13 active ingredients (Figure 1(a)). In total, 50 genes were obtained after transforming with UniProt.

### 3.2. Potential Targets of Shan-Zhu-Yu in the Treatment of Depression

In the present study, 9721 genes were documented as potential targets of depression and 48 intersecting genes between Shan-Zhu-Yu and depression were obtained using the Venn online database (Table 1 and Figure 1(b)).

### 3.3. PPI Network Construction and Visualization

**Figure 2:**
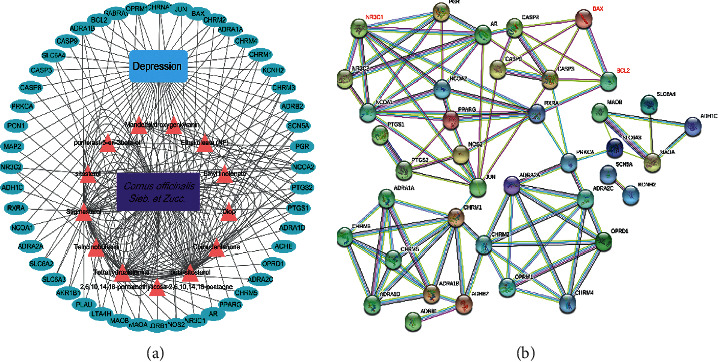
Active ingredients-targets-drug-disease network and PPI network. Purple rectangle node represents drug, orange triangle nodes represent active ingredients, blue rectangle node represents disease, and blue-green oval nodes represent target genes. (a) PPI network. Nodes represent genes, connections represent interactions, and different colored connections represent different types of interactions (b).

The 48 intersecting genes were input into the STRING network. The core genes were screened thoroughly, and 40 genes were identified. Using the Cytoscape software, the results were clearly obtained in the TSV file format (Figure 2(a)), and the PPI network was downloaded (Figure 2(b)). The PPI network revealed that the main active ingredients of Shan-Zhu-Yu in the treatment of depression are sitosterol, beta-sitosterol, telocinobufagin, stigmasterol, DTOP, cornudentanone, 2,6,10,14,18-pentamethylicosa-2,6,10,14,18-pentaene, ethyl linolenate, hydroxygenkwanin, ethyloleate, mandenol, and poriferast-5-en-3-beta-ol.

### 3.4. GO and KEGG Pathway Enrichment Analyses

**Figure 3:**
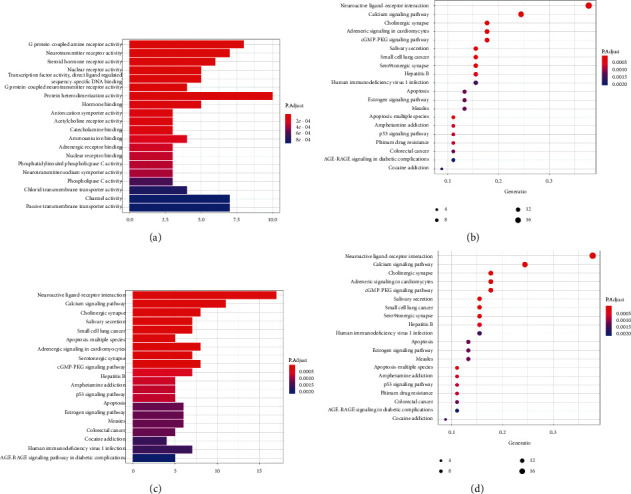
Enrichment analysis of intersecting genes (the top 20 terms of each part are shown). GO enrichment analysis (a). KEGG pathway analysis (b). The higher the column and the redder the color, the more the intersecting genes of enrichment; the sizes of the bubbles are illustrated from big to small in descending order of the number of the intersecting genes involved in the pathways.

To further elucidate the mechanism of drug treatment systematically, enrichment analysis of the 48 intersecting genes was performed using DAVID. The top 20 GO items and KEGG pathways were selected based on *P* values, which represent the degree of enrichment. GO enrichment analysis describes the biological mechanisms of the drug in the treatment of diseases from three aspects: biological processes, molecular functions, and cellular components. Regarding biological processes, Shan-Zhu-Yu might affect the activity of G protein-coupled amine receptors, neurotransmitter receptors, steroid hormone receptors, nuclear receptors, G protein-coupled neurotransmitter receptors, protein heterodimerization, an acetylcholine receptor, and phosphatidylinositol phospholipase C in the treatment of depression by acting on hormone, catecholamine, and adrenergic receptors as well as nuclear receptor binding (Table 2 and Figure 3(a)). In the pathway analysis, 59 signal pathways were identified, which were mainly enriched in neuroactive ligand-receptor interaction, calcium, cGMP-protein kinase G, apoptosis, estrogen, p53, and advanced glycation end products-receptor for advanced glycation end products (Figure 3(b)).

### 3.5. Molecular Docking Analysis

**Figure 4:**
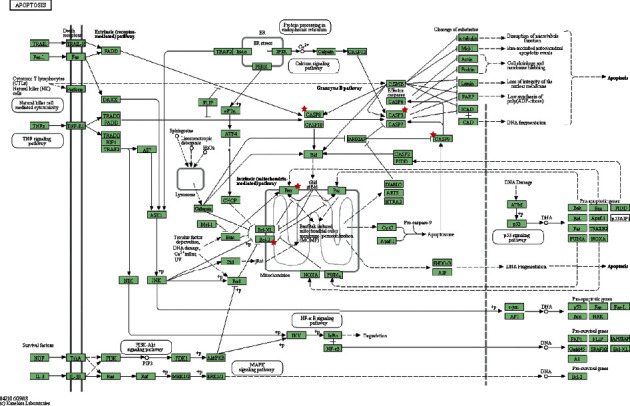
Apoptosis signaling pathway. The genes with a five-pointed star are potential target for Shan-Zhu-Yu in treating depression predicted by network pharmacology.

**Figure 5:**
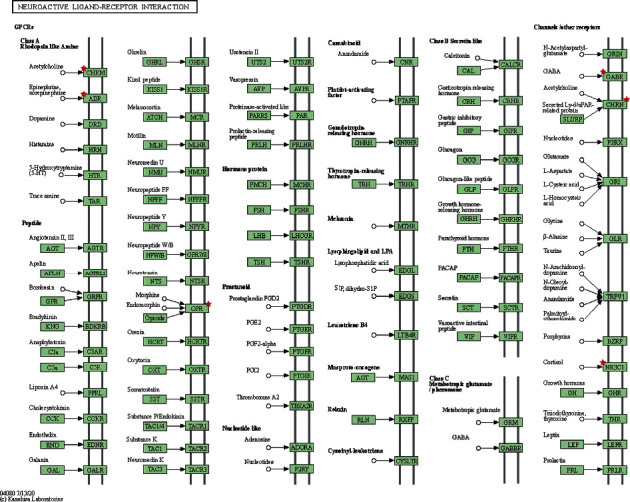
Neuroactive ligand-receptor interaction signaling pathway. The genes with a five-pointed star are potential targets for Shan-Zhu-Yu in treating depression predicted by network pharmacology.

The most important signaling pathway of Shan-Zhu-Yu in the treatment of depression was determined to be apoptosis. The genes enriched in the apoptosis pathway (Figure 4, granted permission already) include *Bax, Bcl-2,* and *caspase-*3; the major effective ingredient may be beta-sitosterol. Among the core genes, *NR3C1* (glucocorticoid receptor (GR); Figure 5, granted permission already) may regulate the apoptosis pathway; the ingredient that regulates *NR3C1* is telocinobufagin.

**Figure 6:**
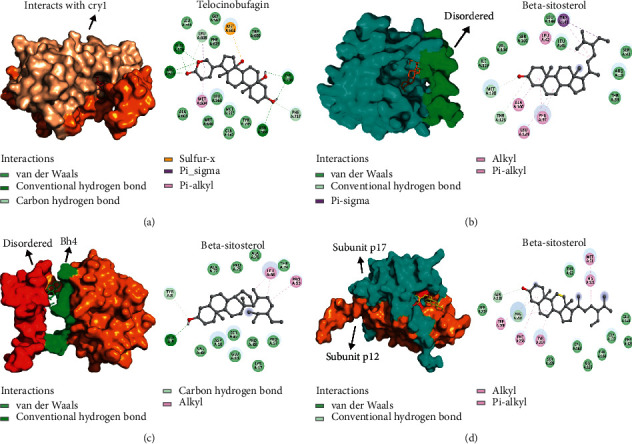
Docking conformation of active ingredients and potential targets. Telocinobufagin and NR3C1 (a); beta-sitosterol and Bax (b). The secondary structure of the green region is random coils, near which small molecules are bound; Bcl-2 (c); beta-sitosterol and caspase-3 (d). The cyan and brown parts are subunit p17 and subunit p12 domains, respectively. The red part is the two key amino acid residues, His121 and 163Cys, in the active center of caspase-3.

Molecular docking (Figure 6) was performed to study the interactions between active ingredients and target genes. The lower the energy of the molecule, the more stable the conformation.

We searched the UniProt Database and obtained the NRLBD domain of *NR3C1* protein, in which the gray part of the structure interacts with CRY1. The optimal conformation of telocinobufagin binds to the active pocket of the nuclear receptor ligand-binding domain of *NR3C1*; the lowest binding free energy is −9.43 kcal/mol, and the main forces involved are van der Waals forces, hydrophobic forces, and carbon-hydrogen bonds.

The optimal conformation of beta-sitosterol binds with *Bax*; the secondary structure of the green region is a randomly coiled domain, and small molecule is bound in the pocket near the random coil domain of the *Bax* protein; the lowest binding free energy is −7.9 kcal/mol, and the main forces are van der Waals forces, hydrophobic forces, and carbon hydrogen bonds.

The docking results show that the optimal conformation of the beta-sitosterol is bound between the random coil and BH4 of the *Bcl-2* protein, the lowest binding free energy is −8.21 kcal/mol, and there are alkyl hydrophobic forces between Pro88, Pro59, and Leu86 and beta-sitosterol. Hydroxyl hydrogen atoms form a conventional hydrogen bond with Gly8 and carbon-hydrogen bond with Tyr9. Thr74, Ser87, Asp10, and Val89 form van der Waals forces with each other.

The crystal structure of *caspase-3* was downloaded from UniPort. The optimal conformation of beta-sitosterol was bound between p12 and p17 subunits, and the lowest binding free energy was −7.16 kcal/mol (Table 3).

## 4. Discussion

In the present study, network pharmacology methods were used to explore possible targets and mechanisms of Shan-Zhu-Yu in the treatment of depression. Using TCMSP, 13 active ingredients of Shan-Zhu-Yu with OB ≥30% and DL ≥0.18 were identified, and using GeneCards and OMIM, 9721 disease-related genes were identified. Using a Venn tool, 48 drug-disease intersecting genes were identified, which are the potential targets for Shan-Zhu-Yu in the treatment of depression. A PPI network was constructed with 40 genes, and active ingredients that may affect depression were determined. GO enrichment analysis identified 97 genes, and KEGG enrichment analysis identified 59 pathways involved although only the top 20 are shown owing to space limitations. Genes involved in the neuroactive ligand-receptor interaction signaling pathway and apoptosis signaling pathway are likely related to the pathological mechanism of depression; therefore, genes in these pathways are key targets for the treatment of depression. Additionally, the connection between these pathways in depression may be a focus in the study of depression [22].

Depression is caused by multiple factors [23], and studying the relationship between factors is essential to clarify the pathogenesis of depression. High levels of glucocorticoids can induce neuronal death, weaken hippocampal neurogenesis, damage the normal function of the HPA axis, and cause depression-like behavior [24], and GR is a major mediator of glucocorticoids. In patients with depression, the expression of GR and GR mRNA in multiple brain regions (especially in the hippocampus) is reduced [25] and *NR3C1* is abnormally methylated [26]. Furthermore, *NR3C1*, which encodes GR, is related to *affective disorders*, and any abnormalities in *NR3C1* affect the function and activity of GR, thereby leading to disorders of the neuroendocrine system [27]. Moreover, *NR3C1* downregulation promotes the expression of micro-RNA-22, which results in the increased expression of downstream genes, namely, Bcl-2-associated agonist of cell death, *Bax*, and *caspase-3*, and decreased expression of *Bcl-2* and *Bcl-xL*, thereby promoting cell apoptosis [22].

In addition, Shan-Zhu-Yu extract can increase the ratio of *Bcl-2/Bax* and reduce the expression of *caspase-3* in the damaged cortex; increase the levels of nerve growth factor and brain-derived neurotrophic factor in the chronic phase; enhance the expression of synaptophysin I, synaptophysin, and postsynaptic density protein 95; inhibit brain trauma apoptosis regulation in the chronic phase [28]; and regulate the level of corticosterone [29]*, all could play a neuroprotective effect*. Molecular docking showed that telocinobufagin and beta-sitosterol, the active ingredients of Shan-Zhu-Yu, can act on *NR3C1*, *Bax*, and *Bcl-2* to treat depression.

Cinnabar (HgS) is a mineral in traditional Chinese medicine for sedation and antianxiety [30, 31]. Shan-Zhu-Yu has various pharmacological effects, including antidepressant effects, when used alone and as a component in antidepressant recipes, such as Liuwei Dihuang pills [13, 15]. Shan-Zhu-Yu could also work with other traditional medicines, such as cinnabar (HgS) to exert better beneficial effects on the brain.

Currently, there are few *in vivo* and *in vitro* studies of Shan-Zhu-Yu in the treatment of depression. Therefore, related research should be developed, which can provide insights into the application of Shan-Zhu-Yu for depression treatment. The results of this study can lay the foundation for research of Shan-Zhu-Yu in treating depression and its mechanism. *Nevertheless, there is also a limitation to this study. Network pharmacology is a research based on database and literature; thus, some action pathways are not selected or some irrelevant pathways are selected and unguided conclusions are obtained owing to the imperfection of selection methods, parameter settings, and calculation models. Overcoming this limitation will render this method more practical, help in better drug mechanism research, and even aid in the development of new drugs and disease treatments.*

## 5. Conclusion

In summary, network pharmacological analysis showed that there are as many as 48 possible targets for Shan-Zhu-Yu in the treatment of depression. The active ingredients, telocinobufagin and beta-sitosterol, may play an important role in the antidepressant effect of Shan-Zhu-Yu via the GR and apoptosis pathway. A signaling pathway comprising *NR3C1* and its downstream genes *Bax*, *Bcl-2*, and *caspase-3* could be one of the possible mechanisms. Therefore, the results of this study provide evidence for follow-up research and a basis for the clinical application of Shan-Zhu-Yu and its prescriptions in the treatment of depression.

## Figures and Tables

**Table 1:** Intersecting genes of Shan-Zhu-Yu in treating depression.

	SZY target genes
Potential genes of depression	PTGS1, PTGS2, NCOA2, PGR, SCN5A, ADRB2, CHRM3, CHRM1, CHRM4, CASP3, NOS2, ADRA1A, CHRM2, ADRA1B, CHRNA2, SLC6A4, OPRM1, GABRA1, BCL2, BAX, CASP9, JUN, CASP8, PRKCA, PON1, MAP2, NR3C2, ADH1C, RXRA, NCOA1, ADRA2A, SLC6A2, SLC6A3, AKR1B1, PLAU, LTA4H, MAOB, MAOA, NR3C1, AR, PPARG, CHRM5, ADRA2C, OPRD1, ACHE, ADRA1D, ADRB1, KCNH2

**Table 2:** Enriched genes in the biological processes.

Description	*P* value	Enriched genes
G protein-coupled amine receptor activity	3.94^*∗*^10^–15^	ADRB2, CHRM3, ADRA1A, CHRM2, ADRA2A, ADRB1, ADRA2C, ADRA1D
Neurotransmitter receptor activity	3.58^*∗*^10^–09^	CHRM3, CHRM1, CHRM2, CHRNA2, OPRM1, GABRA1, ADRB1
Steroid hormone receptor activity	7.58^*∗*^10^–09^	PGR, NR3C2, RXRA, NR3C1, AR, PPARG
Nuclear receptor activity	1.65^*∗*^10^–07^	PGR, RXRA, NR3C1, AR, PPARG
Transcription factor activity, direct ligand regulated sequence-specific DNA binding	1.65^*∗*^10^–07^	PGR, RXRA, NR3C1, AR, PPARG
G protein-coupled neurotransmitter receptor activity	3.64^*∗*^10^–07^	CHRM3, CHRM2, OPRM1, ADRB1
Protein heterodimerization activity	8.30^*∗*^10^–07^	ADRA1A, ADRA1B, BCL2, BAX, JUN, RXRA, ADRA2A, ADRB1, PPARG, ADRA2C
Hormone binding	2.87^*∗*^10^–06^	CHRM3, CHRNA2, NR3C1, AR, ACHE
Anion: cation symporter activity	4.31^*∗*^10^–06^	SLC6A4, SLC6A2, SLC6A3
Acetylcholine receptor activity	4.31^*∗*^10^–06^	CHRM3, CHRM2, CHRNA2
Catecholamine binding	4.31^*∗*^10^–06^	ADRB2, ADRA2A, ADRA2C
Ammonium ion binding	9.19^*∗*^10^–06^	CHRM3, CHRNA2, SLC6A4, ACHE
Adrenergic receptor binding	1.32^*∗*^10^–05^	ADRA2A, ADRB1, ADRA2C
Nuclear receptor binding	1.58^*∗*^10^–05^	NCOA2, RXRA, NCOA1
Phosphatidylinositol phospholipase C activity	1.87^*∗*^10^–05^	CHRM3, CHRM1, CHRM5
Neurotransmitter: sodium symporter activity	2.20^*∗*^10^–05^	SLC6A4, SLC6A2, SLC6A3
Phospholipase C activity	3.87^*∗*^10^–05^	CHRM3, CHRM1, CHRM5
Chloride transmembrane transporter activity	5.44^*∗*^10^–05^	SLC6A4, GABRA1, SLC6A2, SLC6A3
Channel activity	6.57^*∗*^10^–05^	SCN5A, KCNH2, CHRNA2, OPRM1, GABRA1, BCL2, BAX
Passive transmembrane transporter activity	6.68^*∗*^10^–05^	SCN5A, KCNH2, CHRNA2, OPRM1, GABRA1, BCL2, BAX

**Table 3:** Molecular docking results.

Compound	Compound 2D structure	Target and PDB ID	Structure with initial ligand	Grid box size	Affinity (kcal/mol)
Telocinobufagin (CAS: 472-26-4)	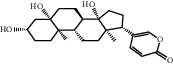	NR3C1 (1NHZ)	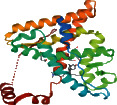	80^*∗*^80^*∗*^80	−9.43
		Bax (homology modeling)	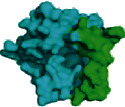	90^*∗*^90^*∗*^90	−7.9
Beta-sitosterol (CAS: 83-46-5)	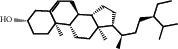	Bcl-2 (homology modeling)	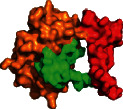	70^*∗*^70^*∗*^70	−8.21
		Caspase-3 (1NMS)	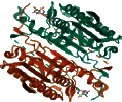	90^*∗*^90^*∗*^90	−7.16

## Data Availability

The data used to support the findings of this study are included within the article.
